# Dietary Cold Pressed Watercress and Coconut Oil Mixture Enhances Growth Performance, Intestinal Microbiota, Antioxidant Status, and Immunity of Growing Rabbits

**DOI:** 10.3390/ani8110212

**Published:** 2018-11-17

**Authors:** Mahmoud Alagawany, Mohamed E. Abd El-Hack, Adham A. Al-Sagheer, Mohammed A. Naiel, Islam M. Saadeldin, Ayman A. Swelum

**Affiliations:** 1 Poultry Department, Faculty of Agriculture, Zagazig University, Zagazig 44511, Egypt; dr.mahmoud.alagwany@gmail.com (M.A.); dr.mohamed.e.abdalhaq@gmail.com (M.E.A.E.-H.); 2 Animal Production Department, Faculty of Agriculture, Zagazig University, Zagazig 44511, Egypt; mohammednaiel.1984@gmail.com; 3 Department of Animal Production, College of Food and Agriculture Sciences, King Saud University, P.O. Box 2460, Riyadh 11451, Saudi Arabia; isaadeldin@ksu.edu.sa; 4 Department of Physiology, Faculty of Veterinary Medicine, Zagazig University, Zagazig 44511, Egypt; 5 Department of Theriogenology, Faculty of Veterinary Medicine, Zagazig University, Zagazig 44511, Egypt

**Keywords:** cold pressed oil, growth, antioxidant, immunity, intestinal microbiota, growing rabbits

## Abstract

**Simple Summary:**

This study focused on using watercress oil and/or coconut oil as growth promotors in intensive rabbit production to promote growth and development while guarding the body against pathogens. From the perspectives of both health and economics, the results showed that several benefits including improvement in growth, feed utilization, antioxidant status, and immunity, and reduction in pathogenic cecal bacteria could be gained by adding 1 g coconut oil + 1 g watercress oil or 0.5 g coconut oil + 1.5 g watercress oil/kg to the diets of rabbits.

**Abstract:**

The present study assessed the effect of dietary supplementation with coconut oil (CNO), watercress oil (WCO), and their mixture as promoters of growth, antioxidant status, immunity, and intestinal microbiota in growing rabbits. A total of 120 rabbits were distributed into six groups (20 rabbits/group) receiving a basal diet without supplementation (G1) or diet supplemented with 2 g CNO (G2), 2 g WCO (G3), 0.5 g CNO plus 1.5 g WCO (G4), 1 g CNO plus 1 g WCO (G5), or 1.5 g CNO plus 0.5 g WCO/kg (G6). Live body weight and feed conversion ratio were significantly higher in the G4 and G5 groups than in the other groups. Superoxide dismutase activity and reduced glutathione concentration were significantly improved in the CNO or WCO diets. Supplemental CNO plus WCO at all tested levels produced the best lysozyme and complement 3 activities. Cecal lactobacilli, coliform, *Enterobacteriaceae*, and *Clostridium* spp. populations were lower in the group who received the 1 g CNO + 1 g WCO/kg diet than that in the control group. Dietary supplementation of 1 g CNO + 1 g WCO or 0.5 g CNO + 1.5 g WCO/kg had the potential to improve growth, feed utilization, antioxidant status, and immunity, and reduce cecal pathogenic bacteria in rabbits.

## 1. Introduction

The use of natural growth promoters in rabbit rations is a widely used strategy to enhance feed efficiency. Phytogenic supplements are products of plant origin that are included in animal feed to amend livestock performance [1]. The addition of herbs or their extracts and active molecules to animal and poultry feed may have beneficial effects such as the improvement of feed intake, stimulation of digestive enzyme secretion, and activation of immune function as well as antiviral, antibacterial, antioxidant, and anthelminthic effects [2]. Some of the medical effects of herbal plants are related to their secondary metabolites such as phenolic compounds, saponins, and essential/necessary oils [3,4].

Currently, cold pressed oils have become widely and commercially offered. These oils were known to contain higher concentrations of phenolic compounds. Therefore, cold pressed seed oils may be used in diverse diets to provide health benefits. Cold pressed seed oils with high content of natural antioxidants may play a key role in decreasing health disorders and risks of chronic ailments. Phenolic compounds are known as potent bioactive molecules with a remarkable antioxidant effect. This effect is chiefly attributed to the probable redox activity, which can efficiently help in deactivating free radicals, chelating certain metals such as copper and iron, as well as quenching ROS [5,6].

Coconut oil (CNO) has been included as a feed additive owing to its beneficial effects, such as antioxidant, anti-inflammatory, and antibacterial activities [7,8]. Caproic acid, capric acid, tocotrienols, and lauric acid are natural antioxidants that constitute the major components of CNO. These bioactive components efficiently scavenge reactive oxygen species (ROS), which play a key function in atherosclerosis, aging, diabetes mellitus, and cancer [9]. Moreover, a substantial reduction in feed efficiency, total cholesterol, serum triglycerides, and low and very low-density lipoproteins as well as glucose levels has been found in diets supplemented with 1% CNO [10].

*Nasturtium officinale* R.Br. (watercress) is a perennial herb and belongs to the mustard family, which is native to North America and Eurasia. It is a detoxifying plant that is rich in several vitamins such as vitamin A, C, E, B1, and B2 as well as minerals including iodine, phosphorus, and iron [11]. Watercress has health benefits that include its effects as a strengthener of immunity, and positive effects have been reported in cancer research [12]. Watercress contains 95% water and has low concentrations of protein, fiber, carbohydrates, and fat. It is particularly rich in menadione and contains large quantities of riboflavin, vitamin B6, vitamin A, vitamin C, calcium, and manganese. Additionally, 100 g of watercress provides 11 calories [13]. Saturated oil in CNO makes up approximately 90%, with medium-chain fatty acids (MCFA) representing 60% of this oil with chain length ranging from 6 to 12 carbon atoms [14], which are incorporated into the hepatic circulation without re-esterification in the digestive tract cells [15]. MCFA are partially autonomous of the transport mechanism of the carnitine compound into the liver mitochondria and are entirely oxidized for energy release [16]. On the contrary, generally, long-chain fatty acids present in feeds are assimilated into chylomicrons following absorption in the intestinal cells because these fatty acids are exposed to re-esterification and then proceed to the blood via the lymphatic system [15]. Rego Costa et al. [17] stated that most long-chain fatty acids are stored in the body fat (adipose tissue). Despite the palatability of watercress for livestock, there are limited studies on the practical usage of watercress or its derivatives such as watercress oil (WCO) as natural feed additives.

There is a vital need to use effective and economic alternatives or combinations of diverse alternatives to improve the performance, productivity, and health of livestock and poultry. Thus, in the present study, the effect of CNO and WCO as feed additives in growing rabbit diets on growth performance, carcass parameters, antioxidant indices, immunity, and intestinal microbiota was examined.

## 2. Materials and Methods

### 2.1. Animals, Diets, and Experimental Protocol

The present study was undertaken at the Rabbit Research Farm, Faculty of Agriculture, Zagazig University, Zagazig, Egypt. All protocols involving animals were approved by the Ethics of Animal Use in Research Committee of Zagazig University. All experimental procedures were performed based on the Directive 2010/63/EU of the European Parliament and the Council of 22 September 2010 on the safety of animals used for scientific studies. A total of 120 New Zealand White (NZW) rabbits (male, 5 weeks old) with an average weight of 511.50 ± 13.44 g were included in the study. Rabbits were randomly allocated into six groups, of two animals per cage, each including ten replicates being the cage considered as a productive unit. Animals were reared in similar sanitary and managerial circumstances, and nourished to meet their nutritional needs based on the methodology described by De Blas and Mateos [18]. Rabbits were kept in cages (stainless steel, 45 cm high × 35 cm wide × 50 cm long) provided with feeders and automatic nipple drinkers. All animals were housed in artificially illuminated and naturally ventilated room. The average values of day light length and ambient temperature during the experimental period were 11.24 h and 20.46 °C, respectively. The present study continued for 8 weeks to be ended at 13 weeks of age. The experimental groups were as follows: G1 was fed the basal diet without any supplementation. G2 and G3 were fed the basal diet supplemented with 2 g CNO/kg diet and 2 WCO/kg diet, respectively. G4 was fed the basal diet supplemented with 0.5 g CNO + 1.5 g WCO/kg diet. G5 was fed the basal diet supplemented with 1 g CNO + 1 g WCO/kg diet. G6 was fed the basal diet supplemented with 1.5 g CNO + 0.5 g WCO/kg diet. CNO and WCO were obtained from Free Trade Egypt Company, Behira, Egypt. All tested diets were formulated, pelleted, and stored at the farm during the experiment (Table 1). Rabbits were fed ad libitum during the entire experimental period.

### 2.2. Gas Chromatography/Mass Spectrometry Examination of CNO and WCO

The major and minor components of the essential oils (CNO and WCO) were identified and measured using gas chromatography/mass spectrometry (Agilent Technologies 6890 series, Wilmington, DE, USA) based on the methodology described by Adams [19].

### 2.3. Antibacterial Activity of Cold Pressed Oil

The bacterial strains used in the present study were acquired from the Egyptian Culture Collection (MERCIN, Ain Shams University, Cairo, Egypt). The antibacterial activity of CNO and WCO against Gram-negative bacteria (i.e., *Salmonella enteritidis* PT4 and *Escherichia coli* ATCC 8739) and Gram-positive bacteria (i.e., *Staphylococcus aureus* ATCC 6538 and *Listeria monocytogenes* Scott A) was determined by the hole plate diffusion method [20].

### 2.4. Growth Performance and Carcass Measurements

Live body weight (LBW), body weight gain (BWG), feed intake (FI), and feed to gain ratio (FCR) were determined based on the methodology described by Alagawany et al. [21]. To determinate LBW all rabbits were weighed at the start of the trial and then every four weeks throughout the experimental duration. FI was measured, every four weeks, by weighing and quantifying the residual amounts of feed then subtracting them from the presented prior to offering the new ones. At the termination of the experiment (13 weeks of age), five animals per group were randomly chosen, weighed and slaughtered to determine all carcass traits. After the complete bleeding, animals were skinned, the viscera are removed, and then the carcasses were weighed. Hot carcass weights (the main body, liver, head, kidneys, heart, lungs, and other total edible parts) and dressing percentage were measured by dividing hot carcass weights on pre-slaughter weight and expressed as a percentage [22]. Carcass yield percentage was calculated by dividing carcass weight after removal of internal organs on pre-slaughter weight and expressed as a percentage. Liver, kidneys, spleen, heart, and lungs were also weighed and expressed as grams per kilogram of pre-slaughter weight.

### 2.5. Antioxidant Indices Assay in Serum

At the end of the experiment, blood samples were aseptically collected from slaughtered animals (5 rabbits/group). The samples were kept to clot and centrifuged for 10 min at 4000 rpm and sera were stored at −20 °C until subsequent analysis. The serum samples were analyzed to determine the superoxide dismutase (SOD) activity and reduced glutathione (GSH) and malondialdehyde (MDA) levels spectrophotometrically using colorimeteric commercial kits purchased from Biodiagnostic Company (Cairo, Egypt) following the methods of according to Nishikimi et al. [23], Beutler et al. [24], and Uchiyama and Mihara [25], respectively.

### 2.6. Lysozyme Activity and Complement Component 3 Assay

Serum lysozyme activity of the experimental animals in each group was determined using the turbidometric method [26]. The lysozyme level was obtained from the logarithmic curve using standard lysozyme. Complement component 3 levels in serum were determined using rabbit complement component 3 ELISA Kit from MyBiosource.com (Catalogue number: MBS701356).

### 2.7. Microbiological Analysis

#### 2.7.1. Feed

At end of the experiment, five dietary samples (approximately 25 g) were randomly taken directly from five different feeders per group, transported in a stomacher bag and then homogenized for 3 min with 225 mL of sterile saline peptone water (SPW). A 10-fold serial dilution was obtained from all samples and used for microbiological investigations. Serial dilutions of SPW per samples were set, and duplicates of 1.0 mL sample of appropriate dilutions were poured on agar plates. Total bacterial count (TBC) was measured in plate count agar for 48 h at 30 °C. Total fungi count (TFC) was enumerated on Rose Bengal Chloramphenicol agar for 5 days at 25 °C. Coliform populations were identified in Violet Red Bile agar at 37 °C following 24 h incubation. *Enterobacteriaceae* were enumerated in Violet Red Bile Glucose agar at 37 °C after 24 h.

#### 2.7.2. Cecal Microflora

At the slaughter time (13 weeks of age), 5 rabbits per treatment were chosen randomly and slaughtered as previously mentioned. From every rabbit, three represented samples of fresh digesta (1–2 g) from the middle part of cecum was retained in bottles under a CO_2_ stream and directly conveyed to the laboratory to determine bacterial populations. Microbial enumerations were performed following the protocols of reference [27].

### 2.8. Statistical Analysis

All statistical analyses were undertaken using the SAS software program [28]. The performance, carcasses, serum constituents, and oxidative status were evaluated with a one-way analysis of variance (with the diet as the fixed factor) using the post-hoc Newman-Keuls test. Significance was established at *p* < 0.05.

## 3. Results

### 3.1. Essential Oils and Bioactive Components in the Oils

The gas chromatography/mass spectrometry analyses results for WCO and CNO with their retention times and peak area percentages are shown in Table 2 and Table 3. The most abundant compounds in WCO were 14-á-H-pregna (44.46%), oleic acid (33.21%), palmitic acid (10.10%), and 2, 4 decadienal (4.83%). WCO also contained 2.85% 2(3H)-furanone, 5-heptyldihydro-(CAS), 2.38% phenol, 2-methoxy-4-(2-propenyl)-(CAS) 0.82% iochiapin B, and 0.28% thymoquinone. In CNO, oleic acid (46.44%) represented the main component, followed by 14-á-H-pregna (24.30%), 2(3H)-furanone, dihydro-5-pentyl (11.36%), lucenin 2 (4.75%), and vanillin (2.53%).

### 3.2. Antibacterial Activities of CNO and WCO against Pathogenic Bacteria

CNO showed antibacterial activity against the examined pathogenic bacteria (TBC, TFC, coliform, and *Enterobacteriaceae*) with inhibition areas of 15.85 ± 0.23, 14.55 ± 0.31, 16.25 ± 0.24, and 15.67 ± 0.21 mm, respectively. WCO showed lower antibacterial activity than that of CNO against the studied pathogenic bacteria with inhibition areas of 12.85 ± 0.19, 12.45 ± 0.16, 13.75 ± 0.28, and 13.65 ± 0.29 mm, respectively. Additionally, in a preliminary trial, the level 2.0% *w/w* of CNO or WCO presented an extreme inhibitory effect on bacterial pathogens. Thus, these concentrations (2.0 g/kg or a combination thereof, i.e., 1.0 + 1.0 or 0.5 + 1.5 or 1.5 + 0.5 g/kg) were chosen for all subsequent trials. The selected concentrations of cold pressed oils (2.0 or 1.0 + 1.0 or 0.5 + 1.5 or 1.5 + 0.5 g/kg) were added to the control diet to check their influence on the reduction of TBC, TFC, coliform, and *Enterobacteriaceae* indicating that there was no significant (*p* < 0.05) alteration between 2.0 g/kg of CNO or WCO and 1.0 + 1.0% levels.

### 3.3. Growth Performance and Carcass Measurements

The effects of dietary cold pressed oil supplements on growth performance of growing rabbits throughout the trial are illustrated in Table 4. LBW at 13 weeks of age was significantly higher in the G4 and G5 groups than that in the other groups (*p* = 0.039). BWG values were significantly higher in both G4 and G5 groups than that of the control group (*p* < 0.05) during 9–13 and 5–13 weeks of age. In the present study, no effect on FI was observed based on different dietary treatments. FCR was statistically (*p <* 0.05) different in rabbits receiving diets containing oils for 9–13 and 5–13 weeks of age. Supplementing the diets with CNO or WCO or its mixtures improved FCR for the overall period (5–13 weeks of age). The lowest value of FCR (*p* = 0.040) was noted in the group fed diet supplemented with 0.5 g CNO + 1.5 g WCO (G4) and 1 g CNO + 1 g WCO/kg diet (G5). As summarized in Table 5, there was no difference among the groups for carcass yield (%), dressing (%), and carcass organs (g/kg slaughter weight). In general, mortality rate was low (one dead animal in G1 and G6 groups) and there was no difference among groups.

### 3.4. Antioxidant Status and Immunity

The influences of dietary cold pressed oils on antioxidant indices involving serum activity of SOD as well as GSH and MDA contents of rabbits are shown in Table 6. SOD activity and GSH level were statistically influenced by the dietary feed additives (*p* < 0.05). SOD activity (*p* < 0.001) and GSH concentration (*p* = 0.033) were significantly improved in the oil-treated groups. Enriching rabbit diets with 1 g CNO + 1 g WCO/kg feed (G5) or 1.5 g CNO + 0.5 g WCO/kg feed (G6) produced higher values of SOD (16.21 and 16.12 U/mL, respectively) than that of the other groups. Also, rabbit fed diets supplemented with 2 g WCO/kg feed (G3) or 1.5 g CNO + 0.5 g WCO/kg feed (G6) showed higher values of GSH (3.27 and 3.58 nmol/L, respectively) than that of the other groups. MDA concentrations were not affected by cold pressed oil supplementation (*p* = 0.197). Supplementing rabbit diets with 0.5 g CNO + 1.5 g WCO/kg feed (G4) or 1 g CNO + 1 g WCO/kg feed (G5) produced lower MDA levels than that in the other groups.

The effects of cold pressed oil supplementation on the immune parameters of rabbits are shown in Table 6. Unique positive effects of cold pressed oil supplements were observed on lysozyme and complement component 3 activities, which were statistically (*p* < 0.05) higher than that of the control group (C1). Supplemental CNO plus WCO at all tested levels produced the highest lysozyme (from 2.42 to 2.70 U/mL) and complement component 3 values (from 105.01 to 110.93 mg/dL).

### 3.5. Effect of Cold Pressed Coconut and Watercress Oils on Microbial Populations

#### 3.5.1. Feed

As shown in Table 7, the TBC, TFC, coliform, and *Enterobacteriaceae* in all treatment groups were significantly altered (*p* < 0.001). The populations of TBC, TFC, and *Enterobacteriaceae* were significantly lower in all treatments than that of the control (*p* < 0.001). However, the maximum reduction of coliform populations and TFC were observed in the 2 g CNO/kg diet (G2). Also, it can be observed that adding the CNO or WCO at 1 g/kg diet (G5) achieved the maximum hindering of the TBC and *Enterobacteriaceae* growth.

#### 3.5.2. Cecum

CNO or WCO levels in the diet influenced the microbial population of the cecum in rabbits (Table 8). The changes in the level of microbial populations in the caecal content of rabbits with different dietary treatments were significantly different (*p* < 0.05). The maximum suppression of TBC populations was found in rabbits who received 1 g CNO + 1 g WCO/kg diet (G5) than that of the control. However, rabbits who received 1.5 g CNO + 0.5 g WCO/kg diet (G6) showed the highest reduction of *Enterobacteriaceae* and coliforms counts. While, the maximum inhibition of lactobacilli and *Clostridium* spp. population was noted in rabbits fed diet enriched with 2 g WCO/kg diet (G2).

## 4. Discussion

In the present study, CNO showed higher antibacterial activity than that of WCO against the tested pathogenic bacteria. Several authors have found that cold pressed oil has antimicrobial activity against Gram-positive and Gram-negative bacteria in vitro, in situ, and in vivo [29,30,31]. Nevin and Rajamohan [7] demonstrated that CNO has antibacterial action against Gram-positive and Gram-negative bacteria. Additionally, CNO has been used as a feed additive owing to its beneficial applications and health benefits such as antioxidant and anti-inflammatory activities [7,8].

The enhancement in LBW, BWG, and FCR with oil mixture (0.5 g CNO +1.5 g WCO and 1 g CNO + 1 g WCO) supplements might be due to providing certain bioactive compounds that improve nutrient digestion and absorption. Similar results were observed by Messens et al. [32], who stated that the antioxidant activities of coconut can strongly change the digestive system condition via antibacterial and antimicrobial effects, which could be mirrored in the enhancement of digestion. In agreeance with our results, El-Abasy et al. [10] revealed lower feed conversion ratios in all coconut treated groups than that in the control group (G1). Langhout [33] reported that these herbs or their derivatives could improve the digestive system of poultry, enhance liver function, and stimulate pancreatic enzymes in the gut. Improvement in nutrient metabolism due to herbal supplementation could improve growth performance [34]. However, the effects of phytogenic additives or their bioactive components are not always detected in growth indices [35]. In fact, numerous in vivo and in vitro studies have used the active components (e.g., tannins, saponins, and flavonoids) that were obtained from these plants, showing that coconut, watercress, or their extracts also have antifungal, anti-inflammatory, antimicrobial, and antioxidant properties [3,10]. Our findings partially disagree with Van Gerwe et al. [36] who postulated that 1% MCFA supplementation increased daily weight gain. Furthermore, these results were verified upon investigation of MCFA in broilers [37] that found that 4% palm oil supplementation as a source of MCFA in diets increased FI.

All carcass parameters were not statistically influenced by dietary treatments in the present study. Our findings are partially in agreeance with Wang et al. [38] who found no significant effects of dietary CNO on poultry carcass traits including dressing and breast percentages. Skřivanová et al. [39] found that carcass yield was not significantly affected by MCFA supplementation in rabbit diets. Similarly, Kovitvadhi et al. [40] mentioned that herbal supplements had no statistical effects on carcass traits or dressing percentage. Furthermore, dressing percentage was not influenced by the addition of herb oils in rabbit diets [41]. Likewise, Al-Sagheer et al. [42] stated that there was no beneficial influence of dietary of phytogenic additives on carcass traits of rabbits exposed to a hot climate. Manesh [43] demonstrated that the addition of watercress extract did not affect breast, liver, and carcass percentages of male broilers. In contrast, rabbits who received 1.5% CNO increased percentages of dressing, trunk, fore parts, hind parts, and total edible parts (*p* ≤ 0.05) [44].

In the present study, the addition of CNO and WCO to the basal diet resulted in a notable elevation of both non-enzymatic (GSH) and enzymatic (SOD) antioxidants. SOD plays a key role in preventing body cells from free radicals and oxidative damage; however, this action requires a supply of definite nutrients in the diet [1]. The detrimental effects of ROS may be inhibited using antioxidant sources that eliminate detoxifying organisms and radicals [45]. These bioactive ingredients may delay the oxidation of proteins or lipids and other nutrients by suppressing the propagation of oxidation reactions [46]. However, there is growing interest in discovering and producing alternative natural antioxidants from medicinal herbs [21,47,48]. Among these plants, coconut and watercress have shown to have higher saturated fatty acids and polyphenolic compounds, as positive correlations have been noted between the content of polyphenolic compounds in herbs and their antioxidant properties [49]. Similar to our results, Aaby et al. [50] and Zeb [51] confirmed that the antioxidant action of WCO may be linked to phenolic molecules such as quercetin and coumaric acid and their derivatives, as well as thymoquinone, caftaric acid, sinapic acid, palmitic acid, oleic acid, and vanillic acid. Dietary supplementation of watercress has shown to reduce the DNA damage and improve the antioxidant activity in the blood [52].

Phytogenic supplements may have multifaceted mechanisms of action, including modifying feed color and flavor, inducing gastric motility, and improving the secretion of digestive enzymes, endocrine function, immune function, and antioxidant status [35,53]. From our results, the addition of natural antioxidants such as herbal additives could be used in the future to enhance the productivity and health status of the rabbit.

Improvement in animal immunity is important to decrease or prevent many infectious ailments via induced immune enhancers and stimulants as the best solutions; herbal oils as feed additives are used in the diets for this purpose. Lysozyme activity is a chief innate immune defense index [54]. Also, complement component 3 contributes to innate immunity and plays a key function in the complement system, which is the main component of the immune system that attacks the pathogenic microbes in cell membrane and improves the efficacy of phagocytic cells and antibodies to clear microbes and damaged cells from an organism [55]. Mugnai et al. [56] has confirmed that serum lysozyme has a synergic action with the serum complement. In the present study, dietary supplementation with WCO, CNO, or their mixtures significantly increased both lysozyme and complement component 3 activities. Our results are in agree with those of El-Abasy et al. [10] who found that the supplementation of coconut (2%) played a role in enhancing immune parameters and health. In addition, increased lysozyme activity with supplementation of CNO may indicate improved immunity in rabbits. Herbal supplements are rich in flavonoids content and other bioactive components act as antioxidants and can thereby improve immunity [57]. This may illustrate the influence of cold pressed oils on immune indicators studied in Table 6. CNO contains lauric acid, myristic acid, palmitic acid, caprylic acid, capric acid, oleic acid, stearic acid, and caproic acid. More than 90% of the fatty acids in CNO are saturated and just less than 10% are unsaturated [58]. Saturated fatty acids in CNO have been found to significantly enhance the innate immune responses [59]. Mohiti-Asli and Ghanaatparast-Rashti [60] observed that broilers fed 300 ppm of essential oil had higher immune parameters than those fed a control diet.

In the present study, dietary CNO, WCO, or their mixtures resulted in a delay in the spoilage emergence. In addition, 1 g CNO + 1 g WCO/kg in the diet inhibited the growth of TBC, lactobacilli, coliform, *Enterobacteriaceae,* and *Clostridium* spp. in the caecal content of rabbits. This inhibition could be attributed to the antimicrobial effect of CNO and WCO, which is mostly because of its high phenolic content [7].

Bacteria colonization on mucosal tissues is a vital step in enteric infections. Bacteria must initially attach to the epithelial cells to settle on the mucosal surface. Binding of type 1 fimbriae is one of the main modes for binding to the epithelium surface [61]. It has been reported that the compressed oil acts as the binding sites for Gram-negative bacteria, inhibiting their enterocytes attachment [62]. This is the possible way by which CNO or WCO could reduce bacterial populations in the present study. An incremented digestibility by supplemental CNO or WCO is another way that these oils could reduce pathogenic bacterial colonies in the gastrointestinal tract. In addition, it has been documented that inferior digestibility values and inefficiently digested diets lead to an increase in the proliferation of putrefying bacteria in the hindgut, which increases toxic metabolites such as biogenic amines and ammonia [61]. The antimicrobial activity of natural extracts is closely related to their phenolics content and polyphenols that are considered as potent active compounds with great antimicrobial and antioxidant actions [63]. The phenolic compound may alter the pH gradient and bacterial cells membrane potential causing disturbance of the intracellular ATP content of *Escherichia coli* and *Salmonella* via the collapse of the Gram-negative bacteria outer membrane. Various in vivo studies reported that many cold pressed oils might prohibit the Gram-negative growth bacteria [6,29,30,31]. Conversely, the use of CNO and WCO in the diet led to a decrease in pathogenic bacteria inhabitants. The outcomes from literature revealed that there were no significant effects of cold pressed oils on favorable bacterial population such as lactobacilli in situ [29,30].

## 5. Conclusions

Our results indicated that the addition of cold pressed oils to rabbit diets has the capacity to improve the productive performance and lessen cecal pathogenic bacteria in growing rabbits. The counts of TBC, *Enterococcus*, coliforms, *Clostridium* spp., and TFC in the content of cecum were lower (*p* < 0.05) in rabbits fed diets containing CNO or WCO or their mixtures than that of the control group. Therefore, the diets of growing rabbits could be fortified with CNO or WCO to improve growth, feed utilization, antioxidant status, immunity, and microbiological status of both diets and cecum of rabbits.

## Figures and Tables

**Table 1:** Ingredients and composition of commercial diet for growing rabbits.

Items	Basal Diet
Ingredient (%)	
Soybean meal	15
Wheat bran	25
Berseem hay	30
Barely grain	28
Limestone	1
NaCl	0.5
Premix ^1^	0.5
Total	100
Calculated composition (%)	
Digestible energy, MJ/kg	10.85
Crude protein	17.29
Calcium	0.87
Phosphorus	0.54
Analyzed composition (%, as-DM basis)	
Crude protein	18.38
Ether extract	2.56
Crude fiber	13.70
Neutral detergent fiber	37.92
Acid detergent fiber	21.93
Dry matter	88.06
Organic matter	90.57
Ash	9.43
Nitrogen free extract	55.93

^1^ Each 1 kg of premix (minerals and vitamins mixture) contains vit. A, 20,000 IU; vit. D3, 15,000 IU; vit. E, 8.33 g; vit. K, 0.33 g; vit. B1, 0.33; vit. B2, 1.0 g; vit. B6, 0.33 g; vit. B5, 8.33 g; vit. B12, 1.7 mg; pantothenic acid, 3.33 g; biotin, 33 mg; folic acid, 0.83 g; choline chloride, 200 g.

**Table 2:** Retention time (RT) and peak area (%) of the different compounds found in watercress oil analyzed by gas chromatography/mass spectrometry.

Compound	RT (min)	Peak Area %
14-á-H-pregna	37.02	44.46
Oleic Acid	32.48	33.21
Palmitic acid	28.93	10.10
2,4 Decadienal	14.77	4.83
2(3H)-Furanone, 5-heptyldihydro-(CAS)	15.64	2.85
Phenol, 2-methoxy-4-(2-propenyl)-(CAS)	15.59	2.38
Isochiapin B	32.73	0.82
Thymoquinone	13.38	0.28

**Table 3:** Retention time (RT) and peak area (%) of the different compounds found in coconut oil analyzed by gas chromatography/mass spectrometry.

Compound	RT (min)	Peak Area %
Oleic Acid	32.93	46.44
14-á-H-pregna	38.52	24.3
2(3H)-Furanone, dihydro-5-pentyl	15.90	11.36
Lucenin 2	41.54	4.75
Palmitic acid	29.05	4.71
Stearic acid	33.12	3.16
Vanillin	16.81	2.53
Fumaric acid, eicosyl 2-hexylester	43.46	1.29

**Table 4:** Effects of treatments on growth performance of NZW growing rabbits at 13 weeks of age (n = 20/group).

Item	G1	G2	G3	G4	G5	G6	SEM ^1^	*p*-Value ^2^
Live body weight (g)								
week 5	524	493	511	515	508	518	13.44	0.993
week 9	1341	1350	1429	1355	1444	1325	37.95	0.672
week 13	2007 ^b^	2011 ^b^	2063 ^b^	2357 ^a^	2350 ^a^	1984 ^b^	49.92	0.039
Body weight gain (g)								
week 5–9	816 ^a,b^	857 ^b^	918 ^a,b^	839 ^a,b^	935 ^a^	807 ^a,b^	25.66	0.033
week 9–13	666 ^b^	660 ^b^	633 ^b^	1002 ^a^	906 ^a^	658 ^b^	33.12	0.001
week 5–13	741 ^b^	758 ^b^	776 ^b^	921 ^a^	921 ^a^	732 ^b^	26.25	0.041
Feed intake (g)								
week 5–9	80.37	74.18	72.97	72.71	80.38	71.57	1.47	0.311
week 9–13	122.57	107.83	119.98	119.11	124.80	119.65	2.26	0.351
week 5–13	101.47	91.00	96.48	95.91	102.59	95.61	2.24	0.090
Feed conversion ratio (g feed/g gain)								
week 5–9	3.42	2.71	2.53	2.74	2.61	2.72	0.21	0.332
week 9–13	5.16 ^a^	4.57 ^a^	5.30 ^a^	3.57 ^b^	3.85 ^b^	5.09 ^a^	0.35	0.008
week 5–13	4.29 ^a^	3.64 ^b^	3.91 ^a,b^	3.15 ^c^	3.23 ^c^	3.90 ^a,b^	0.20	0.040

Different superscripts (^a^ and ^b^) within 1 row are significantly different (*p* < 0.05). G1: control, G2: 2 g coconut oil, G3: 2 g watercress oil, G4: 0.5 g coconut oil + 1.5 g watercress oil, G5: 1 g coconut oil + 1 g watercress oil, G6: 1.5 g coconut oil + 0.5 g watercress oil; ^1^ SEM = Standard error of the mean; ^2^ Overall treatment *p*-value.

**Table 5:** Effects of treatments on carcass traits (as % of pre-slaughter Weight, SW) of NZW growing rabbits at 13 weeks of age (n = 5/group).

Item	G1	G2	G3	G4	G5	G6	SEM ^1^	*p*-Value ^2^
Carcass yield, %	52.23	51.22	52.75	53.80	50.31	52.21	0.37	0.095
Heart, g/kg SW	2.73	2.83	2.79	2.87	2.50	3.72	0.15	0.277
Kidneys, g/kg SW	9.46	12.05	10.40	11.49	9.61	10.01	0.33	0.119
Liver, g/kg SW	37.21	34.70	44.92	39.67	32.40	32.04	1.88	0.367
Spleen, g/kg SW	0.57	0.78	0.73	0.46	0.70	0.52	0.18	0.197
Lungs, g/kg SW	11.26	9.35	7.99	6.83	7.83	8.77	0.56	0.318
Giblets, %	6.12	5.97	6.68	6.13	5.30	5.50	0.19	0.423
Dressing, %	58.35	57.20	59.94	59.94	55.62	57.72	0.46	0.058

G1: control, G2: 2 g coconut oil, G3: 2 g watercress oil, G4: 0.5 g coconut oil + 1.5 g watercress oil, G5: 1 g coconut oil + 1 g watercress oil, G6: 1.5 g coconut oil + 0.5 g watercress oil; ^1^ SEM = Standard error of the mean; ^2^ Overall treatment *p*-value.

**Table 6:** Effects of treatments on antioxidant and immune parameters of NZW growing rabbits at 13 weeks of age (n = 5/group).

Item	G1	G2	G3	G4	G5	G6	SEM ^1^	*p*-Value ^2^
Oxidative status ^3^								
SOD (U/mL)	5.82 ^d^	8.04 ^c^	9.60 ^b,c^	10.87 ^b^	16.21 ^a^	16.12 ^a^	0.97	<0.001
GSH (nmol/L)	1.51 ^b^	2.81 ^a,b^	3.27 ^a^	2.90 ^a,b^	3.11 ^a,b^	3.58 ^a^	0.20	0.033
MDA (nmol/L)	9.90	8.29	8.51	6.68	7.51	8.56	0.36	0.197
Immune parameters								
Lysozyme activity (U/mL)	1.21 ^c^	1.91 ^b^	2.26 ^a,b^	2.43 ^a^	2.42 ^a^	2.70 ^a^	0.17	0.024
Complement 3 (mg/dL)	81.34 ^c^	90.03 ^b^	93.25 ^b^	105.78 ^a^	105.01 ^a^	110.93 ^a^	2.91	0.003

Different superscripts (^a^, ^b^, ^c^, and ^d^) within the same row are significantly different (*p* < 0.05). G1: control, G2: 2 g coconut oil, G3: 2 g watercress oil, G4: 0.5 g coconut oil + 1.5 g watercress oil, G5: 1 g coconut oil + 1 g watercress oil, G6: 1.5 g coconut oil + 0.5 g watercress oil; ^1^ SEM = Standard error of the mean; ^2^ Overall treatment *p*-value; ^3^ SOD: superoxide dismutase, GSH: reduced glutathione, MDA: malondialdehyde.

**Table 7:** Effects of treatments on the average of total bacterial count, *Enterobacteriaceae*, coliform populations, and total fungi count, in experimental diets (n = 5/group).

Item	G1	G2	G3	G4	G5	G6	SEM ^1^	*p*-Value ^2^
Microbiological count (Log CFU/g)								
Total bacterial count	4.53 ^a^	4.41 ^b^	4.41 ^b^	4.43 ^b^	4.25 ^c^	4.41 ^b^	0.02	<0.001
*Enterobacteriaceae*	2.68 ^a^	2.14 ^d^	2.28 ^c,d^	2.39 ^b,c^	2.22 ^d^	2.52 ^b^	0.04	<0.001
Coliforms	2.28 ^a^	1.86 ^c^	2.12 ^a,b^	1.99 ^b,c^	2.11 ^a,b^	2.09 ^a,b^	0.03	<0.001
Total fungi count	2.32 ^a^	1.11 ^b^	1.14 ^b^	1.18 ^b^	1.12 ^b^	1.13 ^b^	0.10	<0.001

Different superscripts (^a^, ^b^, ^c^, and ^d^) within the same row are significantly different (*p* < 0.05). G1: control, G2: 2 g coconut oil, G3: 2 g watercress oil, G4: 0.5 g coconut oil + 1.5 g watercress oil, G5: 1 g coconut oil + 1 g watercress oil, G6: 1.5 g coconut oil + 0.5 g watercress oil; ^1^ SEM = Standard error of the mean; ^2^ Overall treatment *p*-value.

**Table 8:** Effects of treatments on cecal microbiota (total bacterial count, lactobacilli count, *Enterobacteriaceae*, coliform, and *Clostridium* spp.) in NZW growing rabbits (n = 15/group).

Item	G1	G2	G3	G4	G5	G6	SEM ^1^	*p*-Value ^2^
Microbiological count (Log CFU/g)								
Total bacterial count	8.56 ^a^	8.16 ^d^	8.28 ^c^	8.31 ^b^	8.09 ^e^	8.27 ^c^	0.03	<0.001
Lactobacilli	8.36 ^a^	8.03 ^b^	8.05 ^b^	8.06 ^b^	8.11 ^b^	8.14 ^b^	0.03	<0.001
*Enterobacteriaceae*	6.46 ^a^	6.35 ^c^	6.33 ^c^	6.42 ^b^	6.27 ^d^	6.19 ^e^	0.02	<0.001
Coliforms	5.55 ^d^	5.54 ^d^	5.60 ^c^	5.65 ^b^	6.51 ^a^	5.44 ^e^	0.08	<0.001
*Clostridium* spp.	4.58 ^a^	4.13 ^b^	4.18 ^b^	4.12 ^b^	4.19 ^b^	4.15 ^b^	0.04	<0.001

Different superscripts (^a^, ^b^, ^c^, ^d^, and ^e^) within the same row are significantly different (*p* < 0.05). G1: control, G2: 2 g coconut oil, G3: 2 g watercress oil, G4: 0.5 g coconut oil + 1.5 g watercress oil, G5: 1 g coconut oil + 1 g watercress oil, G6: 1.5 g coconut oil + 0.5 g watercress oil; ^1^ SEM = Standard error of the mean; ^2^ Overall treatment *p*-value.
